# A novel acid catalyst based on super/subcritical CO_2_-enriched water for the efficient esterification of rosin

**DOI:** 10.1098/rsos.171031

**Published:** 2018-07-04

**Authors:** Dan Zhou, Linlin Wang, Xiaopeng Chen, Xiaojie Wei, Jiezhen Liang, Dong Zhang, Guoxin Ding

**Affiliations:** 1 School of Chemistry and Chemical Engineering, Guangxi University, Nanning 530004, People's Republic of China; 2 Guangxi Key Laboratory of Petrochemical Resources Processing and Process Intensification Technology, Guangxi University, Nanning 53004, People's Republic of China

**Keywords:** super/subcritical CO_2_–H_2_O, green catalytic esterification, rosin, kinetics, thermodynamics

## Abstract

Rosin esters are widely applied as masticatory substances and beverage stabilizers, while classical acid-catalysed processes will lead to metal residue or environmental issues. Super/subcritical CO_2_–enriched high temperature liquid water (HTLW) as a green acid catalyst in the esterification reaction of rosin with glycerol was investigated. The pH of CO_2_–H_2_O binary system, as calculated based on gas–liquid equilibrium, charge balance and chemical equilibrium equations, ranged from 3.49 to 3.70 depending on the reaction conditions, indicating effective acid catalysis. Response surface methodology experiments showed the optimum conditions were 3.5 h, 3.9 MPa CO_2_ pressure, a rosin-to-glycerol molar ratio of 1.32 and 269°C, and an enhanced esterification yield of 94.74% was achieved, which was superior to that obtained using a ZnO catalyst. It was found that the esterification kinetics was a pseudo first-order reaction, and the enthalpy and entropy of activation were calculated using the Arrhenius–Polanyi equation. The presence of super/subcritical CO_2_-enriched HTLW catalyst can decrease the activation energy and significantly accelerate the reaction rate.

## 1. Introduction

Pine resin is a renewable biological resource secreted by pine trees, and gum rosin can be obtained from this material by distillation at 170–180°C [1]. Resin acids are the main chemical constituents of rosin, accounting for approximately 85.63% to 88.72% [2]. In addition, rosin contains lesser amounts of nonacidic compounds [2]. Resin acids have cyclic structures, with both conjugated and nonconjugated carbon–carbon double bonds and carboxy groups [3]. As a result, several different methods can be used to chemically modify rosin. Esterified rosin resulting from reactions with polyalcohols, including glycerol (1,2,3-propanetriol) and pentaerythritol, is the most important product obtained from this material [4]. Compared with the original rosin, rosin esters exhibit good oxidation resistance and reduced brittleness, low acid values, high thermal stability, high softening points and light colour, and can be widely applied as coating and adhesives, solder fluxes, neutralizers, masticatory substances, base beverage stabilizers, slow-release coating materials and film forming agents [1,5,6]. Additionally, in recent years, rosin esters have been used for a naturally derived excipient in drug delivery systems [6].

Glycerol has a high viscosity and high boiling point, and its applications include cosmetics, textiles and foods, as well as tobacco, leather, pharmaceutical, and pulp and paper products [1,4]. Glycerol production has rapidly increased over the past several years owing to the increased production of biodiesel. However, requirements for glycerol have not kept pace with the supply, such that the market has become saturated and there has been a dramatic drop in price [1]. As a result, there is currently significant interest in expanding the applications of glycerol in various fields and developing new high value products based on this resource [4].

Resin acids have a tricyclic phenanthrene structure that results in high steric hindrance, such that there is a high activation energy for esterification reactions. For this reason, the esterification of rosin has to be carried out at elevated temperatures and using a highly active catalyst [3]. Initially, alkaline catalysts such as CaO, Ca(OH)_2_ and LiOH were used for the esterification of rosin, although these have been largely abandoned because they affect the colour of the rosin ester [5]. Protonic acid catalysts have been reported, including sulfuric and phosphoric acids, but these can lead to corrosion of equipment and have associated environmental concerns [5]. As a substitute, solid acid catalysts such as ZnO, ZnCl_2_, ZnSO_4_ and Zn(OAc)_2_ have recently been reported for the esterification of rosin [7–9]. However, ZnO is difficult to separate from the product and cannot be reused, especially in food grade rosin ester products where there may be issues with residual heavy metal ions [9,10]. Thus, there is a requirement for research regarding the green catalytic conversion of rosin to esters.

In the open literature, it can be found that the esterification of rosin can be also performed with lipase [11] and with ion exchange resins [12]. In recent years, super/subcritical CO_2_-enriched high temperature liquid water (HTLW) binary systems have been widely applied as green, acidic catalytic media to enhance the rates of hydrolysis reactions [13,14]. Fu *et al*. [15] assessed the effects of CO_2_ on the hydrolysis of phenylacetonitrile in HTLW and found that, because the base-catalysed mechanism was dominant under these conditions, CO_2_ did not effectively promote the hydrolysis. Hunter & Savage [16] demonstrated that CO_2_ addition effectively increases the reaction rate during dibenzyl ether hydrolysis in HTLW, and estimated that the pH value of the CO_2_-enriched HTLW system ranges from 3.5 to 5.6 at 150–300°C and a CO_2_ pressure of 10 MPa.

There have been only a few studies regarding the use of super/subcritical CO_2_-enriched HTLW as an acid catalyst for esterification. Catalytic esterification is not only an indispensable aspect of organic synthesis, but also a potentially important technology for processing biomass resources. The present study therefore aimed to seek an effective way using super/subcritical CO_2_-enriched HTLW as an acid catalyst for the esterification of biomass resources.

It is obvious that the application of the non-toxic, non-flammable and inexpensive reagent CO_2_ in conjunction with HTLW for the acceleration of acid catalysis would be environmentally advantageous compared with employing mineral acids. In this work, CO_2_ dissolved in water generated as a by-product of esterification formed carbonic acid and this compound was employed instead of a more traditional catalyst. The pH value of the CO_2_–H_2_O binary system under different reaction conditions during the rosin esterification was calculated using charge-balance and chemical equilibrium equations. Besides, the effects of temperature, CO_2_ pressure, and rosin-to-glycerol ratio (*n*_rosin_ : *n*_glycerol_) on the reaction progress were investigated and optimized using response surface methodology (RSM). The relative activities obtained using CO_2_ addition and a standard ZnO catalyst were assessed. The kinetics of the esterification, and thermodynamics of the formation of the activated complex were examined both with and without CO_2_ addition.

## 2. Material and methods

### 2.1. Materials

Technical-grade rosin (acid value 160.64 mg KOH g^−1^) was purchased from Guangxi Wuzhou Pine Chemicals (Guang-Xi, China). Glycerol (pharmaceutical grade) was obtained from the Guangxi Lantian Equipment (Guang-Xi, China). Analytical reagent grade ZnO (purity ≥99.0%, white crystalline powder, specific density 5.606 g cm^−3^, particle size 0.1 µm) was purchased from the Guangdong Guanghua Technology (Guang-Dong, China). Carbon dioxide (purity greater than 99.0%) was supplied by the Guangxi Guoxin Gas (Guang-Xi, China). Tetrahydrofuran, used as the mobile phase for the gel permeation chromatography (GPC) analysis of rosin and glycerides, was obtained from the Tianjin Fuyu Fine Chemical (Tianjin, China).

### 2.2. Experimental set-up and procedure

Esterification reactions were performed in a 2-l stainless steel autoclave (Dalian Jingyi Autoclave, Dalian, China) equipped with a paddle agitator, cooling coil and electrical heating mechanism. In addition to the electrical heating mechanism, the reaction temperature was also controlled with a water bath (Shanghai Bo Xu Industrial, Shanghai, China) capable of maintaining set temperatures within ±2°C. Glycerol was added to the reaction via a plunger pump (J-SX2/50, Zhejiang High-Pump Technology, Zhejiang, China). A stainless steel tubing connection and valve were fitted to the reactor to allow CO_2_ to be introduced from a gas cylinder while preventing the backflow of the rosin and glycerides.

Esterification reactions were performed by first loading the autoclave with rosin and then heating the autoclave to melt the rosin. When the rosin was almost completely molten, the heating was shut off and the reactor was closed and sealed. Air was subsequently pumped out of the reactor to obtain an absolute pressure of approximately 0.05 MPa, after which the reactor was filled with CO_2_ to a pressure of 0.7 MPa. These conditions were maintained for 10 min to ensure the removal of any air in the system. Following this, the CO_2_ was removed from the autoclave by again applying a vacuum and the reactor was re-charged with CO_2_ to a pressure of 0.3 MPa. This cycle was repeated three times. Thereafter, the autoclave was charged with CO_2_ to obtain a pressure ranging from 3.0 to 6.0 MPa, agitation speed was commenced at 400 r.p.m., the cooling water system was started up and heating of the reactor resumed. Reaction temperatures ranged from 250°C to 270°C; when the required reaction temperature was close, the desired amount of glycerol was added and then the stirring rate was increased to 600 r.p.m. Considering that esterification was a reversible reaction, the mixture was allowed to react for a fixed period of time, after which water was continually released from an outlet from that point to an absolute pressure of approximately 0.1 MPa, and the reactor was re-charged with CO_2_ to bring the pressure back to the desired value. Each reaction was carried out for 3.5 h, at which point a sample was withdrawn from the reactor to test the acid value.

### 2.3. Sample analysis

The acid value of rosin glyceride must comply with the ASTM-based standard ASTM-D 465-92 [1]. In addition, the degree of rosin esterification was also determined based on the rosin conversion. The rosin conversion was calculated as follows:

2.1 $$\text{Rosin conversion}=\frac{1-\text{the acid number of rosin glyceride}}{\text{the acid number of rosin}}\times 100\%.$$

Because high-pressure CO_2_ was employed during the reaction, trace amounts of carbonic acid could remain in the product, thus increasing the acid value of the rosin glyceride. To eliminate this effect, the rosin glyceride was treated by mixing 70.0 g of the product with 80.0 g turpentine in a 500 ml beaker and subsequently heating the mixture to completely melt the product in a 90°C water bath. Subsequently, 150 ml hot water was added to the beaker and the mixture was stirred at 300 r.p.m., while still in the water bath, for 1 h. Following this, the mixture was allowed to stand at room temperature for 30 min to allow the oil and water to stratify. Turpentine and water were separated from the oil phase by reduced pressure distillation, and the remaining rosin glyceride was used to determine the acid value.

### 2.4. Experimental design

RSM is a collection of mathematical and statistical techniques, originally proposed by Box & Wilson [17], that allows the development of experimental designs and the subsequent use of multiple regression to establish relationships between various factors and response values [18–20]. RSM can reduce the number of experimental trials, thus also reducing the time required, and can produce highly precise quadratic regression equations as well as optimize the process conditions.

A Box–Behnken design (BBD) was used to design the experiments and analyse the experimental data, employing the Design Expert 8.0.6 statistical analysis software. A total number of 17 experimental runs including three factors were generated. These three factors were CO_2_ pressure (in range of 3.0–6.0 MPa), rosin-to-glycerol molar ratio (in range of 1.25–1.75), and reaction temperature (in range of 250–270°C), all of which had three levels (−1, 0, and +1) corresponding to low, mid and high values, respectively. Triplicate experiments were carried out at each combination of conditions.

### 2.5. The pH in CO_2_–H_2_O binary system during the esterification reaction

CO_2_ dissolved in water generated as a by-product of esterification formed carbonic acid, which is a weak acid catalyst, in the esterification reaction of rosin using super/subcritical CO_2_-enriched HTLW catalyst, and the pH in CO_2_–H_2_O binary system is an important factor to affect the severity of esterification reaction. Therefore, we should determine the pH in CO_2_–H_2_O binary system obtained from the gas–liquid equilibrium, charge balance and chemical equilibrium equations.

#### 2.5.1. CO_2_–H_2_O binary system gas–liquid equilibrium

Because the CO_2_–H_2_O binary system is a polar, non-ideal system, using the fugacity state equation to determine the gas–liquid equilibrium can generate significant errors and also requires complex calculations. For this reason, the present study employed the virial equation approach over the pressure range of 0.1–10 MPa and temperature range of 200–300°C. The literature [21] shows that the solubility of CO_2_ is small under these conditions, and thus the liquid phase can be considered to represent an infinitely dilute solution, such that the CO_2_ activity coefficient can be approximated as 1. The fugacity can be calculated via the virial equation together with Henry's constant to determine the CO_2_ gas–liquid equilibrium, as follows:

2.2 $$\left\{ \begin{array}{l} {\hat{f}}_{{\mathrm{CO}}_{2}}^{L}={\hat{f}}_{{\mathrm{CO}}_{2}}^{V};{\hat{f}}_{H_{2}O}^{L}={\hat{f}}_{H_{2}O}^{V}\\ {\hat{f}}_{i}^{V}=py_{i}\varphi_{i}^{V};{\hat{f}}_{i}^{L}=f_{i}x_{i}\gamma_{i} \end{array}\right.$$

and

2.3 $$\left\{ \begin{array}{l} f_{{\mathrm{CO}}_{2}}=f_{{\mathrm{CO}}_{2}}^{H}=H_{{\mathrm{CO}}_{2}}^{0}(T,P_{H_{2}O}^{S})\exp\int_{p_{H_{2}O}^{S}}^{p}\frac{{\bar{V}}_{{\mathrm{CO}}_{2}}\;\text{d}P}{RT}\\ f_{H_{2}O}=P_{H_{2}O}^{S}\phi_{H_{2}O}^{S}\exp\int_{p_{H_{2}O}^{S}}^{p}\frac{{\bar{V}}_{H_{2}O}\;\text{d}P}{RT}. \end{array}\right.$$

Here, $\varphi_{{\;}_{i}}^{V}$ is the fractional fugacity coefficient of component *i* in the gas phase (MPa), ${\hat{f}}_{i}^{k}$ the fractional fugacity of component *i* in the *k*-phase (MPa), *γ* the activity coefficient, *P* the pressure (MPa), *R* the universal gas constant, *T* the temperature (K), $H_{C{\text{O}}_{2}}^{0}$ the Henry's constant of CO_2_ at temperature *T* and saturated vapour pressure of water (MPa) obtained using the model of Crovetto [21], ${\bar{V}}_{i}$ the partial molar volume of component *i* in the liquid phase (m^3^ mol^−1^) obtained using an empirical formula [22], $P_{{\text{H}}_{2}\text{O}}^{S}$ the saturated vapour pressure of water (MPa) obtained from fitting with the Antoine formula.

Under these reaction conditions, CO_2_ does not behave as an ideal gas but rather is in a super/subcritical state, and thus it is more accurate to consider the fugacity of CO_2_ in the non-liquid phase [23]. For a binary *i*, *j* gas mixture, the multicomponent virial equation for the fugacity coefficient simplifies to the following:

2.4 $$\ln\;\varphi_{i}^{V}=\frac{P}{RT}(B_{ii}+y_{j}^{2}\delta_{ij})\;\delta_{ij}=2B_{ij}-B_{ii}-B_{jj}.$$

Here, *B_ij_* is the second virial coefficient, which can be expressed using mixing rules proposed by Smith [24]:

2.5 $$B_{ij}=\frac{{\left(V_{ci}^{1/3}+V_{cj}^{1/3}\right)}^{3}}{4(Z_{ci}+Z_{cj})}\left(\left(0.083-\frac{0.422}{T_{r}^{1.6}}\right)+\left(\frac{w_{i}+w_{j}}{2}\right)\;\left(0.139-\frac{0.172}{T_{r}^{4.2}}\right)\right)$$

and

2.6 $$T_{cij}={\left(T_{ci}T_{cj}\right)}^{0.5}(1-k_{ij}),$$

where *V*_c_ is the critical volume, *Z*_c_ the critical compressibility, *T*_r_ the reduced temperature and *w* the acentric factor and *k* is zero in the approximate calculation.

2.7 $$\left\{ \begin{array}{l} x=\frac{yP\varphi_{CO_{2}}^{V}}{H_{CO_{2}}^{0}(T,P_{H_{2}O}^{S})\exp(({\bar{V}}_{CO_{2}}(P-P_{H_{2}O}^{S}))/RT)}\\ 1-y=\frac{(1-x)P_{H_{2}O}^{S}\varphi_{H_{2}O}^{S}}{P\varphi_{H_{2}O}^{V}}\exp\left(\frac{{\bar{V}}_{H_{2}O}(P-P_{H_{2}O}^{S})}{RT}\right) \end{array}\right.$$

where *x* and *y* are the mole fraction of CO_2_ in the liquid phase and gas phase, respectively.

#### 2.5.2. Values of pH

The estimation of pH values (that is, the estimation of [H^+^] in liquid water) involves a charge-balance equation and three chemical equilibrium equations, as shown in equations (2.8) to (2.11). Here, [N] represents the concentration of substance N (mol kg^−1^) and [CO_2_ (aq)] represents the CO_2_ concentration in the liquid phase. Equation (2.8) can be reduced to equation (2.12) for [H^+^] by incorporating the three equilibrium constants *K*_w_, *K*_a1_ and *K*_a2_. In this manner, [H^+^] can be obtained using only these three equilibrium constants and [CO_2_ (aq)].

The charge balance in the CO_2_–H_2_O system provides the following relationship:

2.8 $$[{\text{H}}^{+}]=[\text{O}{\text{H}}^{-}]+[\text{HC}{{\text{O}}_{3}}^{-}]+2[\text{C}{{\text{O}}_{3}}^{2-}].$$

In addition, the chemical equilibrium of water gives the equation

2.9 $$[{\text{H}}_{2}\text{O}]=[{\text{H}}^{+}]+[\text{O}{\text{H}}^{-}]\;K_{w}=\frac{\left[{\text{H}}^{+}][\text{O}{\text{H}}^{-}\right]}{\left[{\text{H}}_{2}\text{O}\right]}.$$

Finally, based on the chemical equilibrium of CO_2_ (including the primary and secondary ionization) we can write the following:

2.10 $$[\text{C}{\text{O}}_{2}(\text{aq})]+[{\text{H}}_{2}\text{O}]=[{\text{H}}^{+}]+[\text{HC}{{\text{O}}_{3}}^{-}]\;K_{a1}=\frac{\left[{\text{H}}^{+}][\text{HC}{{\text{O}}_{3}}^{-}\right]}{\left[{\text{H}}_{2}\text{O}][\text{C}{\text{O}}_{2}(\text{aq})\right]}$$

and

2.11 $$[\text{HC}{{\text{O}}_{3}}^{-}]=[{\text{H}}^{+}]+[\text{C}{{\text{O}}_{3}}^{2-}]\;K_{a2}=\frac{\left[{\text{H}}^{+}][\text{C}{{\text{O}}_{3}}^{2-}\right]}{\left[\text{HC}{{\text{O}}_{3}}^{-}\right]}.$$

During the actual calculation process, it was found that the carbonate ion concentration is very small, and thus the effect of this variable on pH is minimal. Calculating the pH both including and neglecting the secondary ionization, the difference is less than 0.01. For this reason, the secondary ionization can be omitted, giving the expression for [H^+^] concentration in equation (2.12):

2.12 $$[{\text{H}}^{+}]=\frac{K_{w}}{\left[{\text{H}}^{+}\right]}+\frac{K_{a1}}{\left[{\text{H}}^{+}\right]}[\text{C}{\text{O}}_{2}(\text{aq})].$$

The following expression for [CO_2_ (aq)] can also be employed:

2.13 $$[\text{C}{\text{O}}_{2}(\text{aq})]=\frac{1000x}{M_{W}(1-x)+M_{CO_{2}}x}.$$

In this work, the model proposed by Marshall & Franck [25] was used to calculate *K*_w_, and *K*_a1_ was obtained from the model of Van Walsum [26]. Subsequently, equation (2.7) was applied to determine *x*, after which [CO_2_(aq)] was obtained from equation (2.13), where *M* represents the molar mass of the substance in g mol^−1^. This series of calculations requires only the reaction temperature, *T*, the total pressure, *P*, and the CO_2_ pressure, $P_{C{\text{O}}_{2}}$, to be known to calculate the pH value in the liquid phase, as shown in equation (2.14):

2.14 $$\text{pH}=-\text{lo}{\text{g}}_{10}\left[{\left(K_{w}+K_{a1}\frac{1000x}{M_{W}(1-x)+M_{CO_{2}}x}\right)}^{0.5}\right].$$

### 2.6. Kinetic model

This work used super/subcritical CO_2_-enriched HTLW catalyst to replace a more traditional ZnO catalyst as green acidic catalyst to enhance the esterification of rosin, as a result of it, it has only discussed in detail the kinetic model with and without CO_2_ addition. The esterification reaction of rosin and glycerol was performed with and without using the CO_2_-enriched HTLW catalyst over a reaction time of 210 min. Sample aliquots were withdrawn at 0, 5, 10, 15, 25, 35, 45, 55, 65, 75, 90, 120, 150 and 200 min and analysed by GPC to develop a kinetic model and to explore the reaction mechanism. During GPC analyses, unreacted rosin and rosin glyceride products could be separated and identified according to their elution times, as a consequence of their different molecular weights. The rosin concentrations were determined by calibration with standards [1,4]. The rate equation for the esterification of the rosin is as follows:

2.15 $$r=-\frac{\text{d}C_{R}}{\text{d}t}=k_{0}{C_{R}}^{\alpha }{C_{G}}^{\beta }.$$

Ladero *et al*. [4] have reported that the rosin-glycerol system evolved from a biphasic system to a monophasic one, and the solubility of glycerol in rosin increased from 0.05 to 0.45 mol l^−1^ as the temperature was raised from 240°C to 260°C. At higher temperatures, glycerol solubility is constant with increasing temperature and equal to 0.45 mol l^−1^, probably due to the fact that the boiling point of glycerol at atmospheric pressure is 292°C. Moreover, the solubility of glycerol in the mixture of rosin and rosin triglyceride can be expected to be similar. The present work uses a stoichiometric excess of glycerol in the reaction mixtures, that is, the concentration of glycerol is greater than 0.45 mol l^−1^ as the reaction is over. Therefore, it is enough to be considered constant for kinetic analysis [27]. Thus, the rosin esterification reaction rate equation can be simplified as follows:

2.16 $$-\frac{\text{d}C_{R}}{\text{d}t}=k{C_{R}}^{\alpha },k=k_{0}{C_{G}}^{\beta }.$$

Here, *k*_0_, *k* are the rate constant and the apparent rate constant, respectively, min^−1^; *α*, *β* the reaction order; *t* the reaction time in min^−1^; *C*_R_ and *C*_R0_ are the rosin concentrations at time *t* and initial time, respectively, *C*_G_ is the glycerol concentration at time *t*, mol l^−1^.

### 2.7. Thermodynamic model

On the basis of the above kinetic model, the Eyring–Polanyi equation (equation (2.17)) relating the rate constant to temperature can be used to calculate the thermodynamic parameters of formation of the activated complex of the reaction [28–31]. The present study employed the logarithmic form of this equation:

2.17 $$\ln\left(\frac{k}{T}\right)=-\frac{\Delta H}{RT}+\ln\left(\frac{k_{b}}{h}\right)+\frac{\Delta S}{R}.$$

Gibbs free energy changes of formation of the activated complex of the reaction (Δ*G*) could be calculated by applying the thermodynamic relationship in equation (2.18):

2.18 ΔG=ΔH−TΔS.

## 3. Results and discussion

### 3.1. Response surface methodology analysis

#### 3.1.1. Response surface methodology experiments and model fitting

The experimental results based on BBD are presented in table 1. The variance based on data from experiments was analysed and the results are summarized in table 2. The *F* value of 95.08 obtained from these data demonstrates that the model is appropriate. In addition, the lack of fit *F* value of 0.37 shows that the lack of fit is not significant relative to the pure error [18–20]. As a result, the relationship between the acid value and the various reaction factors can be summarized using the polynomial equation given in equation (3.1):

3.1 $$\begin{array}{rl} \text{Acid value} & =9.68+1.12A-0.93B+1.22C-0.15AB-0.81AC\\ & \;-0.25BC+2.47A^{2}-0.075B^{2}+0.71C^{2}. \end{array}$$

**Table 1.** Experimental results of BBD of the binary system CO_2_–H_2_O in esterification reaction.

run	*A*	*B*	*C*	acid value (mg KOH g^−1^)	standard error *δ*%
1	3.0	250	1.50	11.58	3.83
2	6.0	250	1.50	14.21	2.21
3	3.0	270	1.50	10.24	3.42
4	6.0	260	1.25	13.52	1.17
5	3.0	260	1.75	13.81	1.87
6	4.5	260	1.50	9.32	2.58
7	4.5	260	1.50	10.12	3.46
8	4.5	260	1.50	9.82	3.33
9	4.5	250	1.25	9.88	2.67
10	3.0	260	1.25	9.75	4.15
11	4.5	250	1.75	12.81	3.54
12	6.0	260	1.75	14.34	1.78
13	4.5	270	1.25	8.32	2.38
14	6.0	270	1.50	12.27	2.82
15	4.5	260	1.50	9.47	1.68
16	4.5	260	1.50	9.68	3.27
17	4.5	270	1.75	10.24	2.49

**Table 2.** The analysis of variance and statistical model.

source	sum of squares	d.f.	mean square	*F* value	*p*-value Prob > *F*	
model	60.33	9	6.70	95.08	<0.0001	significant
*A*	10.04	1	10.04	142.34	<0.0001	
*B*	6.86	1	6.86	97.35	<0.0001	
*C*	11.83	1	11.83	167.86	<0.0001	
*AB*	0.090	1	0.090	1.28	0.2958	
*AC*	2.62	1	2.62	37.23	0.0005	
*BC*	0.26	1	0.26	3.62	0.0989	
*A*^2^	25.64	1	25.64	363.70	<0.0001	
*B*^2^	0.024	1	0.024	0.33	0.5816	
*C*^2^	2.09	1	2.09	29.70	0.0010	
residual	0.49	7	0.071			
lack of fit	0.11	3	0.036	0.37	0.7815	not significant
pure error	0.39	4	0.097			
cor total	60.83	16				

The coefficient of determination, *R*^2^, is calculated as the ratio of the sum of the squares of regressions to the total sum of squares, and indicates the degree to which the fitting model matches the response. Herein, the predicted *R*^2^ of 0.962 is in reasonable agreement with the adjusted *R*^2^ of 0.982, meaning that 98.2% of the total variations in the response can be explained by the model. These results demonstrate that the relationship between the rosin glyceride acid value and the reaction factors is well described by a quadratic polynomial.

**Figure 1.**
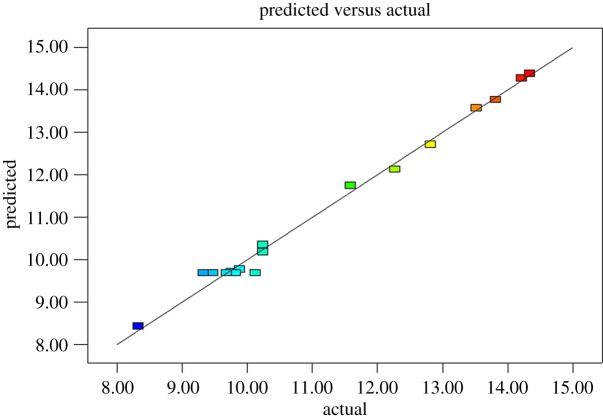
The comparison of the acid value of rosin glyceride ester about prediction and actual values by RSM.

Figure 1 plots the predicted and experimental acid values. From this figure, it is evident that the experimental data are evenly distributed on both sides of the best fit line, while the predicted and experimental values are very close. It is therefore apparent that the BBD can be used to optimize the acid value obtained from the esterification.

#### 3.1.2. Effect of independent variables on the rosin glyceride acid value

A probability > *F* value of less than 0.0500 indicates that the model terms are significant, while a value greater than 0.1000 shows the opposite [28–31]. As can be seen from table 2, terms *A*, *B*, *C*, *AC*, *A*^2^ and *C*^2^ all had a significant effect on the acid value. Because the pH value of the CO_2_–H_2_O system is a function of temperature and CO_2_ pressure, the pH value also affects the acid value.

**Figure 2.**
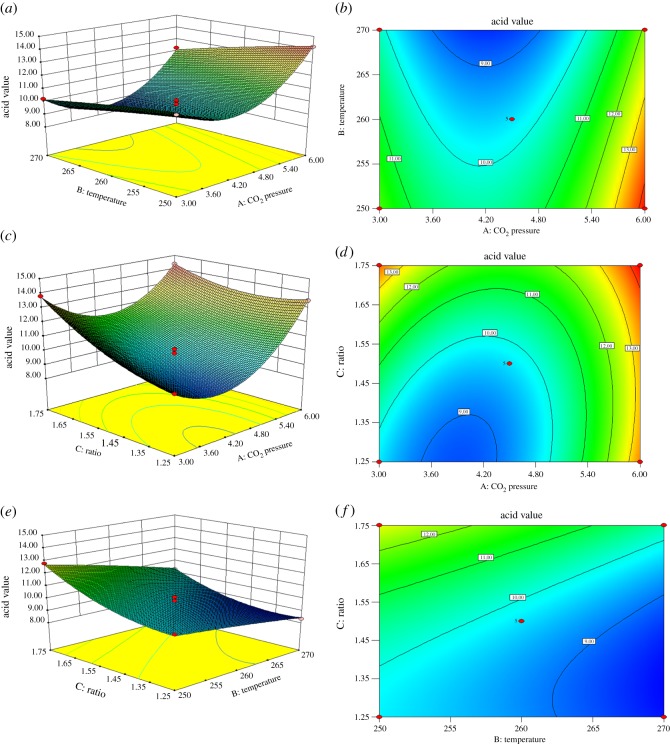
Contour and response surface plots for acid value of rosin glyceride as a function of CO_2_ pressure versus temperature (*a*,*b*), CO_2_ pressure versus rosin-to-glycerol molar ratio (*c*,*d*) and temperature versus rosin-to-glycerol molar ratio (*e*,*f*).

Three-dimensional response surface plots and contour maps (figure 2) were generated using a response surface model (equation (3.1)) to allow visualization of the interactive effects of the three reaction parameters on the acid value. Specifically, the effect of CO_2_ pressure was studied in combination with three different temperatures and reactant ratios. Figure 2*a*,*c* clearly shows that the acid value initially declines then increases. A minimum value of 8.32 mg KOH g^−1^ was obtained upon increasing the CO_2_ pressure from 3.0 to 6.0 MPa, coinciding with a CO_2_ pressure of approximately 4.0 MPa. According to the *F* and *p* values in table 2, the interaction between *A* and *C* (*F *= 37.23 and *p *= 0.0005) had the greatest effect on the acid value. As well, figure 2*e*,*f* shows that applying a lower rosin-to-glycerol molar ratio and a higher temperature reduces the acid value. Under conditions consisting of a CO_2_ pressure of 4.5 MPa, a rosin-to-glycerol ratio of 1.25 and a temperature of 270°C, an acid value of 8.32 mg KOH g^−1^ was obtained. The gradual changes in the acid values in figure 2*b* also demonstrate that *A* and *B* had the least significant interaction (*F *= 1.28, *p *= 0.2958).

#### 3.1.3. Optimization and validation

A final verification trial was performed based on the RSM optimization results, applying the following conditions: 3.9 MPa CO_2_ pressure, a rosin-to-glycerol molar ratio of 1.32 and 269°C. This trial was repeated three times, after which the average acid value (8.45 mg KOH g^−1^) was compared to the optimization result (8.21 mg KOH g^−1^). It is evident that the average of the experimental values is quite close to the optimization value, with an error of just 2.8%, indicating that the optimization was successful. Besides, these conditions give a highest rosin conversion of 94.74%.

#### 3.1.4. Comparative test

From table 3, it can be seen that the rosin and glycerol can only be partially esterified without the addition of the catalyst, such that a high acid value is obtained. The traditional ZnO catalyst greatly increases the rosin conversion and thus reduces the acid value. However, the super/subcritical CO_2_-enriched HTLW system enhances the conversion of rosin to esters to a greater extent than the ZnO, resulting in a low acid value of 8.45 mg KOH g^−1^. Thus, the super/subcritical CO_2_-enriched HTLW system represents a superior acid catalyst for the rosin esterification.

**Table 3.** The effect of different catalysts on the severity of esterification of rosin.

product	catalyst	acid value (mg KOH g^−1^)	esterification rate (%)
rosin (raw material)	—	160.64	—
rosin glyceride	without catalyst	66.54	58.58
rosin glyceride	ZnO	10.23	93.63
rosin glyceride	CO_2_ pressure of 3.95 MPa	8.45	94.74

### 3.2. Analysis of the pH in the CO_2_–H_2_O binary system

The pH values in the CO_2_–H_2_O binary system during the esterification reaction under experimental conditions based on the BBD were calculated using equation (2.14) and are summarized in table 4.

**Table 4.** The pH values of the binary system CO_2_–H_2_O in esterification reaction.

$P_{{\mathrm{CO}}_{2}}$ (MPa)	*P* (MPa)	*y*	*T* (°C)	*Φ*_1_	${\bar{V}}_{1}^{l}$	*x*	*K*_W_	*K*_a1_	pH
3.0	5.3	0.564	270	0.985	9.583 × 10^−3^	0.108	2.425 × 10^−9^	7.083 × 10^−9^	3.70
4.5	5.5	0.818	250	0.973	9.495 × 10^−3^	0.140	2.539 × 10^−9^	1.322 × 10^−8^	3.53
4.5	6.6	0.684	270	0.977	9.583 × 10^−3^	0.143	2.425 × 10^−9^	7.083 × 10^−9^	3.65
3.0	4.5	0.667	250	0.981	9.495 × 10^−3^	0.102	2.539 × 10^−9^	1.322 × 10^−9^	3.58
4.5	6.4	0.708	260	0.975	9.545 × 10^−3^	0.134	2.500 × 10^−9^	9.713 × 10^−9^	3.60
6.0	7.1	0.851	260	0.968	9.545 × 10^−3^	0.166	2.500 × 10^−9^	9.713 × 10^−9^	3.56
4.5	6.2	0.721	250	0.972	9.495 × 10^−3^	0.140	2.539 × 10^−9^	1.322 × 10^−8^	3.53
3.0	5.2	0.573	260	0.983	9.545 × 10^−3^	0.113	2.500 × 10^−9^	9.713 × 10^−9^	3.63
4.5	6.5	0.698	260	0.975	9.545 × 10^−3^	0.139	2.500 × 10^−9^	9.713 × 10^−9^	3.59
6.0	7.5	0.805	270	0.971	9.583 × 10^−3^	0.186	2.425 × 10^−9^	7.083 × 10^−9^	3.61
6.0	7.0	0.855	250	0.965	9.495 × 10^−3^	0.171	2.539 × 10^−9^	1.322 × 10^−8^	3.49
4.5	6.6	0.684	260	0.975	9.545 × 10^−3^	0.139	2.500 × 10^−9^	9.713 × 10^−9^	3.59
3.0	5.3	0.571	260	0.983	9.545 × 10^−3^	0.093	2.500 × 10^−9^	9.713 × 10^−9^	3.66
6.0	7.8	0.774	260	0.967	9.545 × 10^−3^	0.184	2.500 × 10^−9^	9.713 × 10^−9^	3.55
4.5	6.4	0.705	270	0.977	9.583 × 10^−3^	0.138	2.425 × 10^−9^	7.083 × 10^−9^	3.66

**Figure 3.**
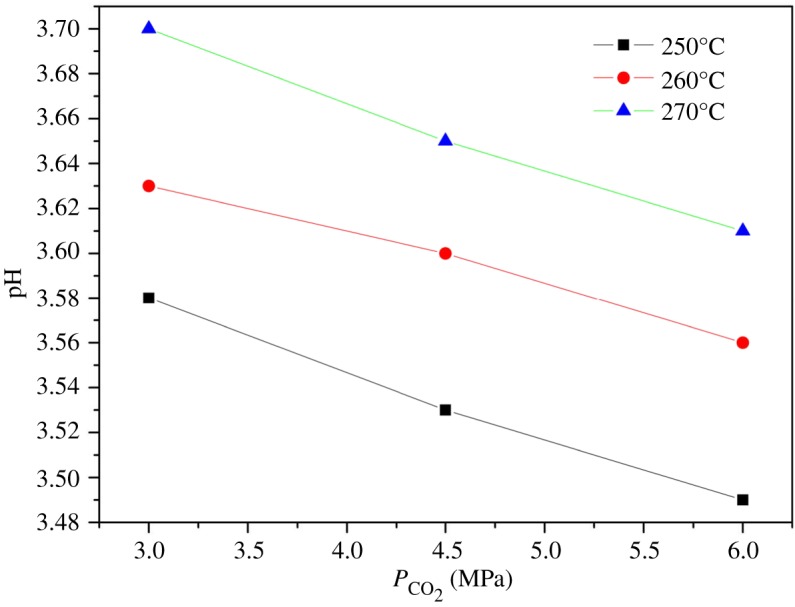
A comparison of the pH value of the binary system CO_2_–H_2_O in esterification reaction at different temperatures, which was calculated by charge-balance equation and chemical equilibrium equations. Solid lines are calculated values and symbols are experimental values for 250°C (filled square), 260°C (filled circle) and 270°C (filled triangle).

These pH values were determined using the gas–liquid equilibrium, charge balance and chemical equilibrium equations at temperatures of 250–270°C and CO_2_ pressures of 3.0–6.0 MPa. The results show that the pH was a function of both temperature and CO_2_ pressure (figure 3), and ranged from 3.49 to 3.70. These values demonstrate that the binary CO_2_–H_2_O system is capable of acting as an effective acid catalysis for the esterification of rosin. Lower temperatures and higher CO_2_ pressures resulted in lower pH values in the CO_2_–H_2_O system, indicating the greater acidity. The solubility of CO_2_ decreases with increasing temperature and increases with increasing CO_2_ pressure. However, low temperatures will also reduce the rosin conversion, while higher pressure will require the use of more expensive equipment, and thus it is important to select the appropriate temperature and CO_2_ pressure for the esterification reactions.

The esterification reaction is typically restricted by the chemical reaction equilibrium, which can lead to poor conversion of rosin to esters. To break the limit of chemical equilibrium, we took the following measures: firstly, water was continually released for a fixed period of time; besides, CO_2_ consumed the water to form carbonic acid; finally, an excess of glycerol was added. The presence of the super/subcritical CO_2_-enriched HTLW catalyst can consume water to promote the reaction to move forward, and improve the reaction rate (figure 7) and reduces the rate of the secondary decarboxylation reaction. In addition, rosin is not miscible with glycerol, which normally results in slow interphase transfer of reactants. The presence of super/subcritical CO_2_ can increase the miscibility of the glycerol and rosin, and influence the physical properties of the glycerol and rosin binary system in esterification reaction, such as reducing the viscosity and improving the diffusion coefficients of the reactants [32].

### 3.3. Analysis of kinetics

**Figure 4.**
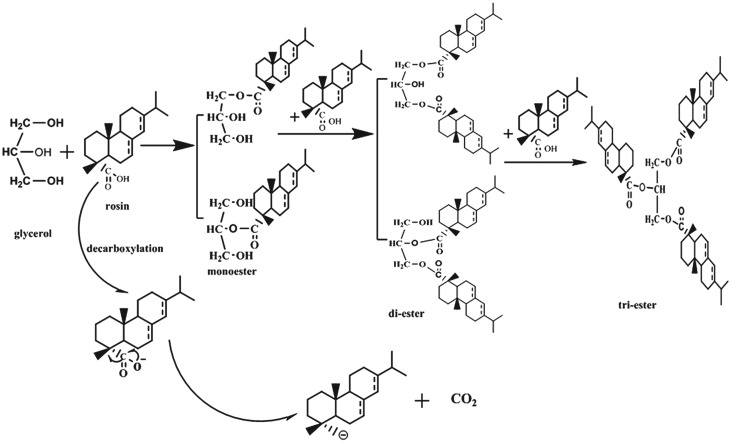
The reaction scheme of esterification and decarboxylation of resin acids.

**Figure 5.**
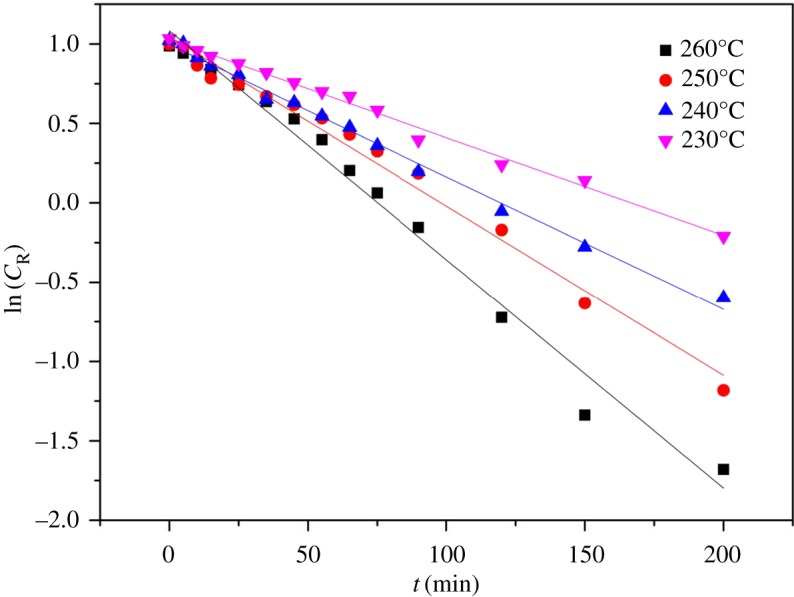
The change of the natural logarithm of rosin concentration with time at different temperatures. Reaction conditions: stirring speed of 600 r.p.m., rosin of 800.0 g, rosin-to-glycerol molar ratio of 1.5, CO_2_ pressure of 4.0 MPa. Solid lines are calculated values and symbols are experimental values for 260°C (filled square), 250°C (filled circle), 240°C (filled triangle) and 230°C (filled inverted triangle).

**Figure 6.**
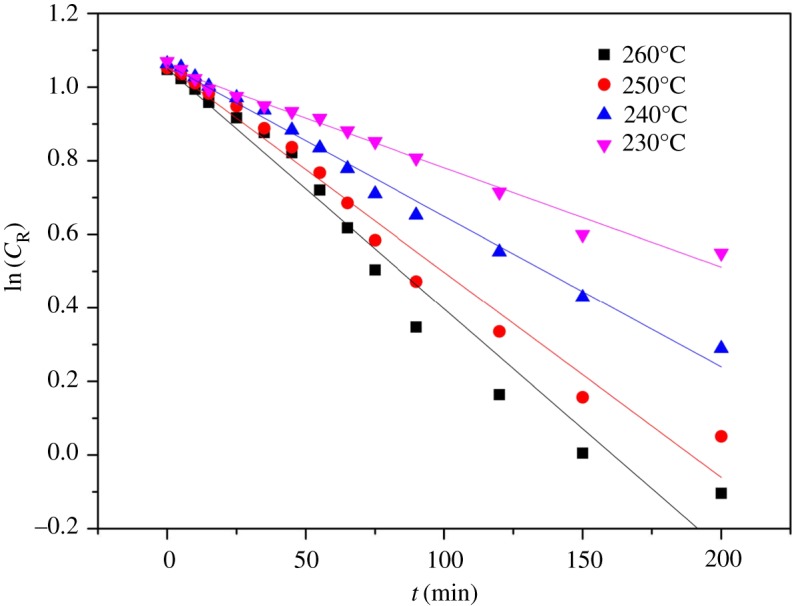
The change of the natural logarithm of rosin concentration with time at different temperatures. Reaction conditions: stirring speed of 600 r.p.m., rosin of 800.0 g, rosin-to-glycerol molar ratio of 1.5, N_2_ pressure of 4.0 MPa. Solid lines are calculated values and symbols are experimental values for 260°C (filled square), 250°C (filled circle), 240°C (filled triangle) and 230°C (filled inverted triangle).

By GPC analysis, rosin glyceride products contained: monoesters, diesters, triesters and a very small amount of deacidification products. A reaction scheme is drawn in figure 4. The rosin reaction order was determined from the experimental concentration data using the Origin software for curve fitting. The rosin concentration data obtained with and without the addition of CO_2_ are shown in figures 5 and 6. As can be seen, there is a clear linear relationship between the natural log of the concentration, ln(*C*_R_), and time, *t*. In addition, regression analysis determined a high value for *R*^2^ (above 0.966). Therefore, the esterification reaction between rosin and glycerol is a pseudo first-order reaction, such that *α* is approximately unity and the slope is approximately equal to the apparent rate constant *k* [27,28,33], as in the rate equation

3.2 $$\ln C_{R}=\ln C_{R,0}-kt.$$

**Figure 7.**
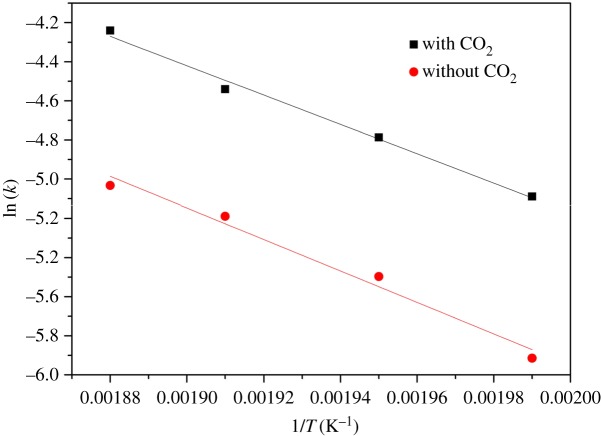
Comparisons of the activation energy and the rate constants for the esterification with CO_2_ addition and without CO_2_ addition at different temperatures. Solid lines are calculated values and symbols are experimental values with CO_2_ addition (filled square) and without CO_2_ addition (filled circle).

The activation energies (*E_a_* and *E_a_*_1_, with and without CO_2_) and frequency factors (*A*_1_ and *A*_2_, with and without CO_2_) were obtained by plotting the experimental rate constants obtained at different temperatures from the Arrhenius equation (equation (3.3)) using the Origin software package (figure 7), and are summarized in table 5. Furthermore, table 5 shows the *k* values obtained at various temperatures in the range of 230–260°C and the *R*^2^ coefficients for experimental trials with and without CO_2_ addition.

3.3 $$\ln k=-\frac{E_{a}}{R}\frac{1}{T}+\ln A.$$

**Table 5.** A comparison of the kinetic parameters for the esterification with CO_2_ addition and without CO_2_ addition.

	*T* (°C)						
parameters	230	240	250	260	*k*_0_ (min^−1^)	*E*_a_ (kJ mol^−1^)	*R*^2^
*k*_1_ (min^−1^)	0.0062 ± 0.0002	0.0083 ± 0.0002	0.0107 ± 0.0004	0.0144 ± 0.0005	1.8993 × 10^4^	62.45 ± 0.78	0.988
*R*^2^	0.992	0.994	0.986	0.984			
*k*_2_ (min^−1^)	0.0027 ± 0.0001	0.0041 ± 0.0001	0.0056 ± 0.0002	0.0065 ± 0.0003	2.5288 × 10^4^	66.88 ± 0.47	0.972
*R*^2^	0.984	0.990	0.976	0.966			

From these data it is evident that the apparent rate constants increased with temperature, and that the apparent rate constants for the reaction with CO_2_ addition were higher than those without CO_2_ addition (applying the same pressure using N_2_ instead). Therefore, the rate of the reaction with CO_2_ addition was faster than that without CO_2_ addition, meaning that the presence of CO_2_ increased the reaction rate. In addition, the activation energy of the reaction using the CO_2_-enriched HTLW catalyst was lower than that without CO_2_. The presence of the CO_2_-enriched HTLW catalyst decreased the activation energy from 66.88 to 62.45 kJ mol^−1^, thus making it easier to perform the esterification [34,35]. Thus, the presence of the CO_2_-enriched HTLW catalyst reduced the energy barrier that was required in the esterification reaction, significantly accelerating the reaction rate. The kinetic parameters for the esterification reaction both with and without the CO_2_-enriched HTLW catalyst at different temperatures in the proposed model met the requirement of an *F*-test at the 95% confidence level, demonstrating a good simulation of the experimental kinetic data.

### 3.4. Analysis of thermodynamics

**Figure 8.**
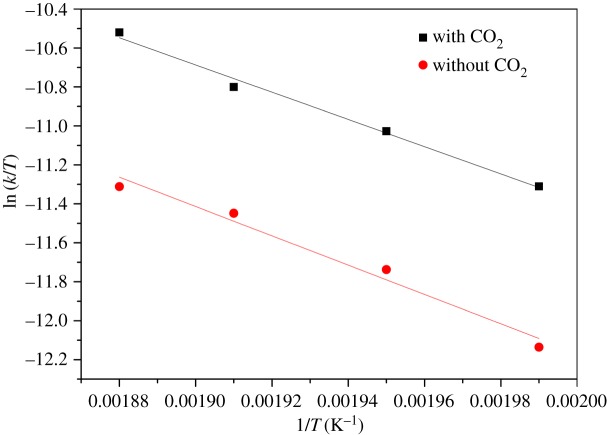
Eyring–Polanyi plots determined the values of enthalpy of activation (Δ*H*) and entropy of activation (Δ*S*) at different conditions. Solid lines are calculated values and symbols are experimental values with CO_2_ addition (filled square) and without CO_2_ addition (filled circle). Best fit: $\ln(k_{1}/T)=-(-\text{6991.36/}T)+2.60,R^{2}=0.988$; $\ln(k_{2}/T)=-(-\text{7525.13/}T)+2.88,R^{2}=0.967$.

The enthalpy changes of formation of the activated complex of the reaction, Δ*H*, and the entropy changes of formation of the activated complex of the reaction, Δ*S*, were obtained by plotting experimental data for rate constants at different temperatures, as shown in figure 8. A plot of ln(*k*/*T*) against 1/*T* yields a slope equal to −Δ*H*/*R*, and Δ*S* can be derived from the intercept: ln(*k_b_*/*h*) + Δ*S*/*R*. The thermodynamic parameters of activation both with and without CO_2_-enriched HTLW catalyst in the proposed model meet the requirement of an *F*-test at the 97% confidence level, indicating that the fitted model was credible. Table 6 provides the values for Δ*H*, Δ*S* and Δ*G*.

**Table 6.** A comparison of the thermodynamic parameters of activation both with CO_2_ addition and without CO_2_ addition at different temperatures.

				Δ*G*/(kJ mol^−1^)			
thermodynamic parameters	Δ*H*/(kJ mol^−1^)	Δ*S*/(kJ K^−1^ mol^−1^)	*R*^2^	230°C	240°C	250°C	260°C
with CO_2_	58.13 ± 0.45	−0.180 ± 0.001	0.988	146.18 ± 0.90	147.93 ± 0.91	149.68 ± 0.92	151.43 ± 0.93
without CO_2_	62.56 ± 0.80	−0.170 ± 0.002	0.967	150.11 ± 1.55	151.85 ± 1.57	153.59 ± 1.58	155.33 ± 1.60

The data in table 6 demonstrate that each of the thermodynamic parameters of activation increased with temperature, and that the values with CO_2_ addition were lower than those without CO_2_ (applying the same pressure using N_2_ instead). The positive Δ*H* values indicate that heat input is required to bring the reactants to the transition state so as to form the products [28,30]. Entropy is a measure of the degree of disorder in a system, and the negative value of Δ*S* shows that the degree of disorder of transition state was lower as compared to reactants in the ground state. The positive Δ*G* indicates that the formation of the activated complex of reaction was unspontaneous and endergonic in nature [28,30]. Moreover, Δ*H* without CO_2_ addition is greater than that with CO_2_ addition, and it is compensated by the greater activation energy for the formation of the activated complex, indicating the CO_2_-enriched HTLW can be an effective acid catalyst [30]. The presence of the CO_2_-enriched HTLW catalyst reduces the required heat input (and thus the energy consumption), promotes the reaction and effectively improves the esterification reaction rate.

## 4. Conclusion

Esterification conditions were optimized by RSM and it was found that the presence of carbonic acid has the effect of increasing the esterification reaction rate. The pH of the binary CO_2_–H_2_O system ranged from 3.49 to 3.70, demonstrating effective acid catalysis for the rosin esterification reaction. Kinetics of the esterification, and thermodynamics properties of the formation of the activated complex were obtained, and the addition of CO_2_ was found to reduce the activation energy and enthalpy of activation while enhancing the reaction rate. These results suggest that carbonic acid may be capable to act as an acid catalyst for the esterification of biomass resources.

## Supplementary Material

Supplementary Material

## Data accessibility

The datasets supporting this article have been uploaded as part of the electronic supplementary material.
