# Supplementary material for: A novel acid catalyst based on super/subcritical CO2-enriched water for the efficient esterification of rosin
Source: R Soc Open Sci. 2018 Jul 4;5(7):171031. doi: 10.1098/rsos.171031 (PMC6083657; doi:10.1098/rsos.171031)
Supplement: Supplementary Material [file rsos171031supp1.pdf]

A novel acid catalyst based on super/sub critical CO_2-water for the efficient esterification of rosin

Dan Zhou[1], Linlin Wang[1,2,*], Xiaopeng Chen[1,2,*], Xiaojie Wei[1,2], Jiezhen Liang[1,2], Dong Zhang[1], Guoxin Ding[1]

[1] *School of Chemistry and Chemical Engineering, Guangxi University, Nanning 530004, PR China*

[2] *Guangxi Key Laboratory of Petrochemical Resources Processing and Process Intensification Technology, Guangxi University, Nanning 53004, PR China*

Supplementary Material

Data processing

1. Conversion

The conversion was calculated as follow.

Conversion = (1- the acid number of rosin glyceride/the acid number of rosin)×100 % (1)

Technical-grade rosin has an acid value of 160.64 mg KOH/g. For an example, a final verification trial was performed based on the RSM optimization results, and the average acid value of 8.45 mgKOH/g was achieved, so the esterification rate was calculated as follow.

Conversion = 1 - 8.45/160.64 = 94.74%

Similarly, we can calculate other values (**Table 1**).

*Corresponding author. Tel.: +86–771–3272702; fax: +86–771–323–3718

E–mail address: wanglinlin1971@sina.com (L. Wang), lilm@gxu.edu.cn (X. Chen).

Table 1 The effect of different catalysts on the severity of esterification of resin acid.

product	catalyst	The acid value /(mgKOH/g)	conversion /%
rosin (raw material)	——	160.64	——
rosin glyceride	without catalyst	66.54	58.58
rosin glyceride	ZnO	10.23	93.63
rosin glyceride	CO_2 pressure of 3.95 MPa	8.45	94.74

2.The pH in CO_2-H_2O binary system during the esterification reaction

2.1 CO_2 -H_2O binary system gas–liquid equilibrium

$$\begin{cases} \hat{f}^{L}_{CO_2} = \hat{f}^{V}_{CO_2} ; \hat{f}^{L}_{H_2O} = \hat{f}^{V}_{H_2O} \\ \hat{f}^{V}_{i} = p y_i \varphi^{V}_{i} ; \hat{f}^{L}_{i} = f_i x_i \gamma_i \end{cases} \tag{2}$$

$$\begin{cases} f_{CO_2} = f^{H}_{CO_2} = H^{0}_{CO_2}(T, P^{S}_{H_2O}) \exp\int_{p^{S}_{H_2O}}^{p} \frac{\overline{V}_{CO_2} dP}{RT} \\ f_{H_2O} = P^{S}_{H_2O} \phi^{S}_{H_2O} \exp\int_{p^{S}_{H_2O}}^{p} \frac{\overline{V}_{H_2O} dP}{RT} \end{cases} \tag{3}$$

Here, Φ_i^k is the fractional fugacity coefficient of component i in the k-phase (MPa), $\hat{f}_i^{\,k}$ the fractional fugacity of component i in the k-phase (MPa), γ the activity coefficient, P the pressure (MPa), R the universal gas constant, T the temperature (K), $H^{0}_{CO_2}(T)$ the Henry's constant of CO_2 at temperature T (MPa) and saturated vapor pressure of water (MPa) obtained using the model of Crovetto (1991), $\overline{V}_i$ the partial molar volume of component i in the liquid phase (m^3/moL) obtained using an empirical formula (Trew et al., 2001), $P^{S}_{H_2O}$ the saturated vapor pressure of water (MPa) obtained from fitting with the Antoine formula.

For a binary i, j gas mixture, the multicomponent virial equation for the fugacity coefficient simplifies to the following.

$$\ln \phi_i = \frac{P}{RT}(B_{ii} + y_j^2 \delta_{ij}) \quad \delta_{ij} = 2B_{ij} - B_{ii} - B_{jj} \tag{4}$$

Here, B_{ij} is the second virial coefficient, which can be expressed using mixing rules proposed by Smith (1975), as below.

$$B_{ij} = \frac{(V_{ci}^{1/3} + V_{cj}^{1/3})^3}{4(Z_{ci} + Z_{cj})}((0.083 - \frac{0.422}{T_r^{1.6}}) + (\frac{w_i + w_j}{2})(0.139 - \frac{0.172}{T_r^{4.2}})) \tag{5}$$

$$T_{cij} = (T_{ci} T_{cj})^{0.5}(1 - k_{ij}) \tag{6}$$

Where V_c is the critical volume, Z_c the critical compressibility, Tr the reduced temperature and w the acentric factor, K is to zero in the approximate calculation.

$$\begin{cases} x = \dfrac{yP\varphi_{CO_2}^V}{H_{CO_2}^0(T, P_{H_2O}^S)\exp(\dfrac{\overline{V}_{CO_2}(P_{CO_2} - P_{H_2O}^S)}{RT})} \\ 1 - y = \dfrac{(1-x)P_{H_2O}^S \varphi_{H_2O}^S}{P\varphi_{H_2O}^V}\exp(\dfrac{\overline{V}_{H_2O}(P - P_{H_2O}^S)}{RT}) \end{cases} \tag{7}$$

Where x and y is the mole fraction of CO_2 in the liquid phase and gas phase, respectively.

2.2 pH values

The estimation of pH values, (that is, the estimation of $[H^+]$ in liquid water) involves a charge–balance equation and three chemical equilibrium equations, as shown in Eqs. (8) to (11). Here, [N] represents the concentration of substance N (mol/kg) and $[CO_2\,(aq)]$ represents the CO_2 concentration in the liquid phase. Eq. (8) can be reduced to Eq. (12) for $[H^+]$ by incorporating the three equilibrium constants K_w, K_{a1} and K_{a2}. In this manner, $[H^+]$ can be obtained using only these three equilibrium constants and $[CO_2\,(aq)]$.

The charge balance in the CO_2-H_2O system provides the following relationship.

$$[H^+]=[OH^-]+[HCO_3^-]+2[CO_3^{2-}] \tag{8}$$

In addition, the chemical equilibrium of water gives the equation below.

$[H_2O]=[H^+]+[OH^-]$ $\quad K_W = \frac{[H^+][OH^-]}{[H_2O]}$ (9)

Finally, based on the chemical equilibrium of CO_2 (included the primary and secondary ionization) we can write the following.

$[CO_2\,(aq)]+[H_2O]=[H^+]+[HCO_3^-]$ $\quad K_{a1} = \frac{[H^+][HCO_3^-]}{[H_2O][CO_2(aq)]}$ (10)

$[HCO_3^-]=[H^+]+[CO_3^{2-}]$ $\quad K_{a2} = \frac{[H^+][CO_3^{2-}]}{[HCO_3^-]}$ (11)

During the actual calculation process, it was found that the carbonate ion concentration is very small, and thus the effect of this variable on pH is minimal. Calculating the pH both including and neglecting the secondary ionization, the difference is less than 0.01. For this reason, the secondary ionization can be omitted, giving the expression for $[H^+]$ concentration in Eq. (12).

$$[H^+] = \frac{K_W}{[H^+]} + (\frac{K_{a1}}{[H^+]}[CO_2(aq)] \tag{12}$$

The following expression for $[CO_2\,(aq)]$ can also be employed.

$$[CO_2(aq)] = \frac{1000\,x}{M_W(1-x) + M_{CO_2}x} \tag{13}$$

In this work, the model proposed by Mashall and Franck (1981) was used to calculate K_w, and K_{a1} was obtained from the model of Van Walsum (2001). Subsequently, Eq. (7) was applied to determine x, after which $[CO_2\ (aq)]$ was obtained from Eq. (13), where M represents the molar mass of the substance in g/mol. This series of calculations requires only the reaction temperature, T, the total pressure, P, and the CO_2 pressure, P_{CO2}, to be known to calculate the pH value in the liquid phase, as shown in Eq. (14).

$$pH = -\log_{10}[(K_W + K_{a1}\frac{1000x}{M_W(1-x)+M_{CO_2}x})^{0.5}] \tag{14}$$

For an example, the conditions were temperature of 270 ℃, CO_2 pressure of 3.0 MPa and the total pressure of 4.59 MPa, and the x and y can be calculated as follow.

1 represents CO_2, and 2 represents H_2O.

(1) The calculation of fugacity coefficient of CO_2

$Y_1 = P_{CO2}/P$ =3/5.32= 0.564

$T_{r1}=T/T_{C1}$= 543.15/304.2 =1.7855 $T_{r2}=T/T_{C2}$=543.15/647.3=0.8391

$B_{11}=V_{C1}\times(0.083\text{-}0.422/T_{r1}^{1.6}+w_1(0.139\text{-}0.172/T_{r1}^{4.2}))\ /Z_{C1}$

$= 343.0657(0.083\text{-}0.422/1.7855^{1.6}+0.225(0.139\text{-}0.172/1.7855^{4.2})) = -19.2223$

$B_{22}=V_{C2}\times(0.083\text{-}0.422/T_{r2}^{1.6}+w_1(0.139\text{-}0.172/T_{r2}^{4.2}))\ /Z_{C2}$

$=343.0657(0.083\text{-}0.422/0.8391^{1.6}+0.225(0.139\text{-}0.172/0.8391^{4.2})) = -134.8739$

T_{r12}= 543.15/(304.2 × 647.3)$^{0.5}$= 1.2240

$B_{12}=(V_{C1}^{1/3}+V_{C2}^{\ 1/3})^3\times(0.083\text{-}0.422/T_{r12}^{1.6}+(w_1+w_1)/2\times(0.139\text{-}0.172/T_{r12}^{4.2}))\ /(4(Z_{C1}+Z_{C2}))$

$=291.7214(0.083\text{-}0.422/1.2240^{1.6}+0.2845(0.139\text{-}0.172/1.2240^{4.2})) = -59.4468$

$\delta_{12}= 2B_{12} - B_{11}- B_{22} =2\times(-59.4468) + 19.2223 + 134.8739 = 35.2026$

$\ln\Phi_1=P(B_{11}+ y_2^2\delta_{12})/\mathrm{RT}$

$= 4.59\times(-19.2223 + (1-0.564)^2\times35.2026)/(8.314\times543.15) =-0.01476$

Therefore, the fugacity coefficient of CO_2 was

$\Phi_1 = 0.985$

(2) The saturated vapor pressure of water

We can obtain the saturated vapor pressure and specific volume of water at different

temperatures from *the water's physical handbook.*

Table 2 The saturated vapor pressure and specific volume of water at different temperatures.

T/℃	250	260	270
saturated vapor pressure/MPa	3.973	4.688	5.499
specific volume/cm^3/g	1.2512	1.2755	1.3023
the density of water p_w/g/cm^3	0.7992	0.7840	0.7679

(3) The calculation of partial molar volume

The partial molar volume of component i in the liquid phase $\overline{V}_i$ (in m^3/moL) obtained using an empirical formula (Trew et al., 2001):

$$\overline{V}_1^l = (6.20M_1 + 0.34)\times(-6.7\times10^{-4}\times T_{r1}+8.1\times10^{-4})/(15(1-T_{r1})^{1.28}) \quad (15)$$

$$= (0.00081-0.00067 \times 1.7855)/(15(1-1.7855)^{1.28})$$

$$= 0.00958 \text{ (m}^3\text{/moL)}$$

(4) Calculation of the Henry's constant of CO_2 at temperature T

From the model of Crovetto (1991), we can calculate the Henry's constant of CO_2 at saturated vapor pressure of water and high temperature.

$$\ln(H^0/bar) = 1713.53(1-T_r)^{1/3}(T/K)^{-1} + 3.875 + 3680.09(T/K)^{-1} - 1198506.1\times(T/K)^{-2} \quad (16)$$

$$\mathrm{Ln}(H^0) = 1713.53\times(1-1.7855)^{1/3}/543.15+3.875+3680.09/543.15-1198506.1/(543.15)^2$$

$$=5.762 \text{ (bar)}$$

Therefore, the Henry's constant of CO_2 at saturated vapor pressure of water can obtain

$H^0 = 31.794$ (MPa)

(6) Calculation of the mole fraction of CO_2 in the liquid phase x

$$x = y_1 p \Phi_1/(H^0 \exp(\overline{V}_1^l (P-P^S{}_2)/RT))$$

$$= 0.564\times5.32\times0.985 /(31.794\times\exp(0.00958\times(5.32-5.499)\times10^6/(8.314\times543.15))$$

=0.108

(7) Calculation of the ion product of water K_W

The empirical model to calculate ion product of water developed by Marshall and Franck (1981) has the following form:

$$\log K_w = A + B/T + C/T^2 + D/T^3 + (E + F/T + G/T^2)\log \rho_w \qquad (17)$$

Where K_w is ion product of water with the unit of $(mol/kg)^2$, ρ_w is the density of water (in g/cm^3)(Table 2), and T is temperature in K. The parameters, A–G, are listed in **Table 3**.

Table 3 Parameters for Eq. (17)

A	-4.098
B	-3245.2
C	2.2362e+5
D	-3.984e+7
E	13.957
F	-1262.3
G	8.5641e+5

Log K_W= $-4.098+-3245.2/543.15+2.2362e+5/543.15^2+-3.984e+7/543.15^3$

$+(13.957+-1262.3/543.15+8.5641e+5/543.15^2)\times LOG10(0.7679)$

$= -11.2306\ (mol/kg)^2$

Therefore, $(K_w)^{1/2} = 2.425E\text{-}09$ (mol/kg)

(8) Calculation of the first dissociation constant K_{a1}

In the study of Van Walsum (2001), the first dissociation constant, K_{a1}, was obtained as follow.

$$pK_{a1} = (2382.3/T) - 8.153 + 0.02194 \times T \qquad (17)$$

$$pK_{a1} = (2382.3/543.15) - 8.153 + 0.02194 \times 543.15 = 8.1498$$

And then K_{a1} =7.083*10^{-9}

(9) Calculation of *pH* values

As Eq. (14), we can calculate the *pH* values at different conditions.

$$pH = -\log_{10}((2.4249E-09 +7.0828E-09\times1000\times0.1576 /(18.0148\times(1-0.1576)+44.009\times0.1576) =3.70$$

Similarly, we can calculate other *pH* values using the same method, and the results were summarized in **Table 4**.

Table 4 The pH values of the binary system CO_2-H_2O in esterification reaction.

P_{CO2}/MPa	P/MPa	y	T/℃	Φ_1	$\overline{V}_1^l$	x	K_W	K_{a1}	pH
3.0	5.3	0.564	270	0.985	9.583E-03	0.108	2.425E-09	7.083E-09	3.70
4.5	5.5	0.818	250	0.973	9.495E-03	0.140	2.539E-09	1.322E-08	3.53
4.5	6.6	0.684	270	0.977	9.583E-03	0.143	2.425E-09	7.083E-09	3.65
3.0	4.5	0.667	250	0.981	9.495E-03	0.102	2.539E-09	1.322E-08	3.58
4.5	6.4	0.708	260	0.975	9.545E-03	0.134	2.500E-09	9.713E-09	3.60
6.0	7.1	0.851	260	0.968	9.545E-03	0.166	2.500E-09	9.713E-09	3.56
4.5	6.2	0.721	250	0.972	9.495E-03	0.140	2.539E-09	1.322E-08	3.53
3.0	5.2	0.573	260	0.983	9.545E-03	0.113	2.500E-09	9.713E-09	3.63
4.5	6.5	0.698	260	0.975	9.545E-03	0.139	2.500E-09	9.713E-09	3.59
6.0	7.5	0.805	270	0.971	9.583E-03	0.186	2.425E-09	7.083E-09	3.61
6.0	7.0	0.855	250	0.965	9.495E-03	0.171	2.539E-09	1.322E-08	3.49
4.5	6.6	0.684	260	0.975	9.545E-03	0.139	2.500E-09	9.713E-09	3.59
3.0	5.3	0.571	260	0.983	9.545E-03	0.093	2.500E-09	9.713E-09	3.66
6.0	7.8	0.774	260	0.967	9.545E-03	0.184	2.500E-09	9.713E-09	3.55
4.5	6.4	0.705	270	0.977	9.583E-03	0.138	2.425E-09	7.083E-09	3.66
